# Important Clues for the Diagnosis of Anti-LGI1-Antibody Autoimmune Encephalitis: A Case Report

**DOI:** 10.7759/cureus.34222

**Published:** 2023-01-26

**Authors:** Mohammad Abu-Abaa, Sindhu Chadalawada, Omar Jumaah, Malik Abubakar, Daniel Landau

**Affiliations:** 1 Internal Medicine, Capital Health Regional Medical Center, Trenton, USA; 2 Neurology, Capital Health Regional Medical Center, Trenton, USA

**Keywords:** hallucination, behavioral change, refractory seizure, hyponatremia, autoimmune encephalitis, lgi1 antibody autoimmune encephalitis

## Abstract

Anti-leucine-rich-glioma-inactivated 1 (LGI1) antibody autoimmune encephalitis is a rare autoimmune encephalitis. We present a 68-year-old female patient who initially presented with episodic confusion, hallucinations, behavioral changes, and unexplained hyponatremia. History was also remarkable for intermittent abnormal movement affecting the left upper extremity and face. She was initially thought to be suffering from dementia and was discharged home. However, progressive symptoms led to her second admission, where evidence of autonomic dysfunction with episodic bradycardia and persistent symptomatic orthostatic hypotension were evident. Generalized cortical hyperexcitability and subclinical seizures were seen. Diagnosis of LGI1 encephalitis was confirmed with a positive Anti-LGI1 antibody in the cerebrospinal fluid, and treatment with intravenous immunoglobulin and steroids improved her cognitive function. This case helps to highlight important features that should raise early clinical suspicion of LGI1 encephalitis, including unexplained progressive hyponatremia, autonomic dysfunction, and frequent refractory seizures. This can lead to earlier recognition of this condition, where earlier implementation of immunosuppressive therapy is linked to better clinical outcomes and brain structural preservation.

## Introduction

Anti- leucine-rich-glioma-inactivated 1(LGI1) antibody autoimmune encephalitis is a rare type of encephalitis. It is the most common autoimmune limbic encephalitis and the second most common after Anti-NMDAR encephalitis [1]. The most prominent clinical features are epilepsy and cognitive decline [2]. It was first described in 2010 [3]. It presents with features of limbic encephalitis, including cognitive decline, behavioral changes, seizure, hyponatremia, and characteristic faciobrachial dystonic seizures (FBDS). It usually presents between 30-80 years of age and is more commonly seen in males in the sixth to seventh decade of life [4].

## Case presentation

A 68-year-old female patient with an unremarkable past medical history presented to the emergency department (ED) for altered mental status. It was reported that for two weeks before her presentation, she developed a few minutes to a few hours of acute confusion, agitation, and visual and auditory hallucination involving her dead relatives. It was also reported that she had intermittent abnormal movement on the left upper extremities and the left side of her face. These were described as a few seconds of mouth angle deviation with elbow flexion of the left upper extremity. Occurrences of wandering in the neighborhood have been reported. She also had difficulty recognizing family members, losing manual dexterity, and dropping objects from her hands. In ED, vital signs included temperatures of 36.2^o^C, heart rate of 75 beats per minute, blood pressure of 145/75 mmHg, respiratory rate of 23 cycles per minute, and SpO_2_ of 89% on room air. Her poor cooperation limited physical examination but showed limited orientation only to herself. Muscle strength, as well as sensory examination, were grossly intact. Deep tendon reflexes were normal.

**Figure 1:**
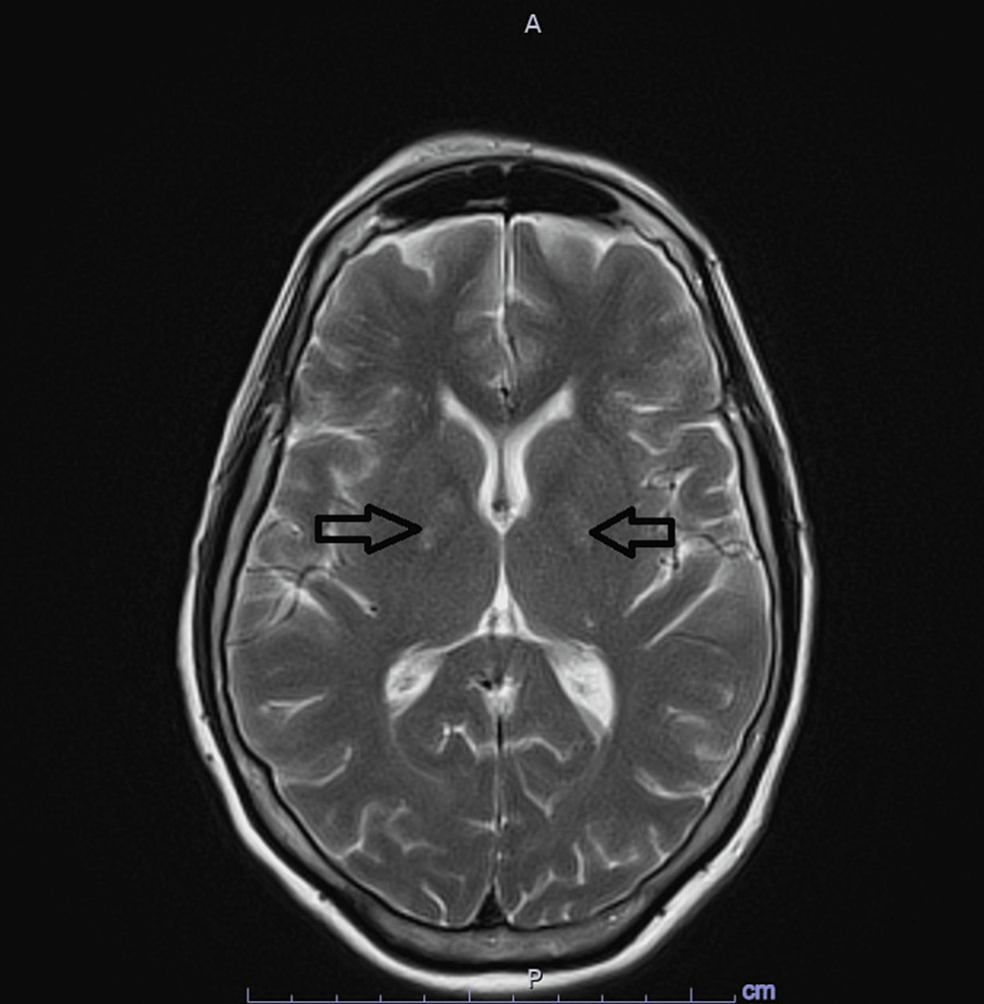
MRI Brain T2 MRI brain showing bilateral hyperintensity seen in globus pallidus (arrows).

Pupils were 2 mm in diameter and reactive bilaterally. No evidence of startle myoclonus nor dystonic movements were seen. Impairment of short and long-term memory as well as concentration was evident. Basic lab work was remarkable only for mild hyponatremia at 134 mmol/L (Reference 137-145 mmol/L). Computed tomography (CT) head was unremarkable. Alcohol level and urine drug screen were negative. No evidence of infection, folate, vitamin B12 deficiency, or hypothyroidism was noted. Magnetic resonance imaging (MRI) brain showed T2 hyperintensity in bilateral globus pallidus and minimal fluid attenuation inversion recovery (FLAIR) series hyperintensity in pons and white matter (Figure 1). Electroencephalography (EEG) showed no epileptic discharges but showed mild generalized slowing. Ultimately, symptoms were attributed to possible dementia, and the patient was discharged home.

**Figure 2:**
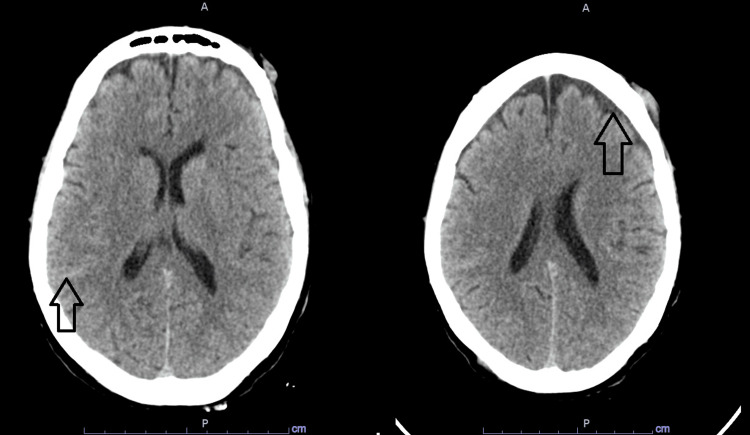
CT Head CT head showing mixed density left-sided frontal subdural hematoma (arrow on the right) and hyperdensity in the right posterior sulci suggestive of subarachnoid hemorrhage (arrow on the left).

**Figure 3:**
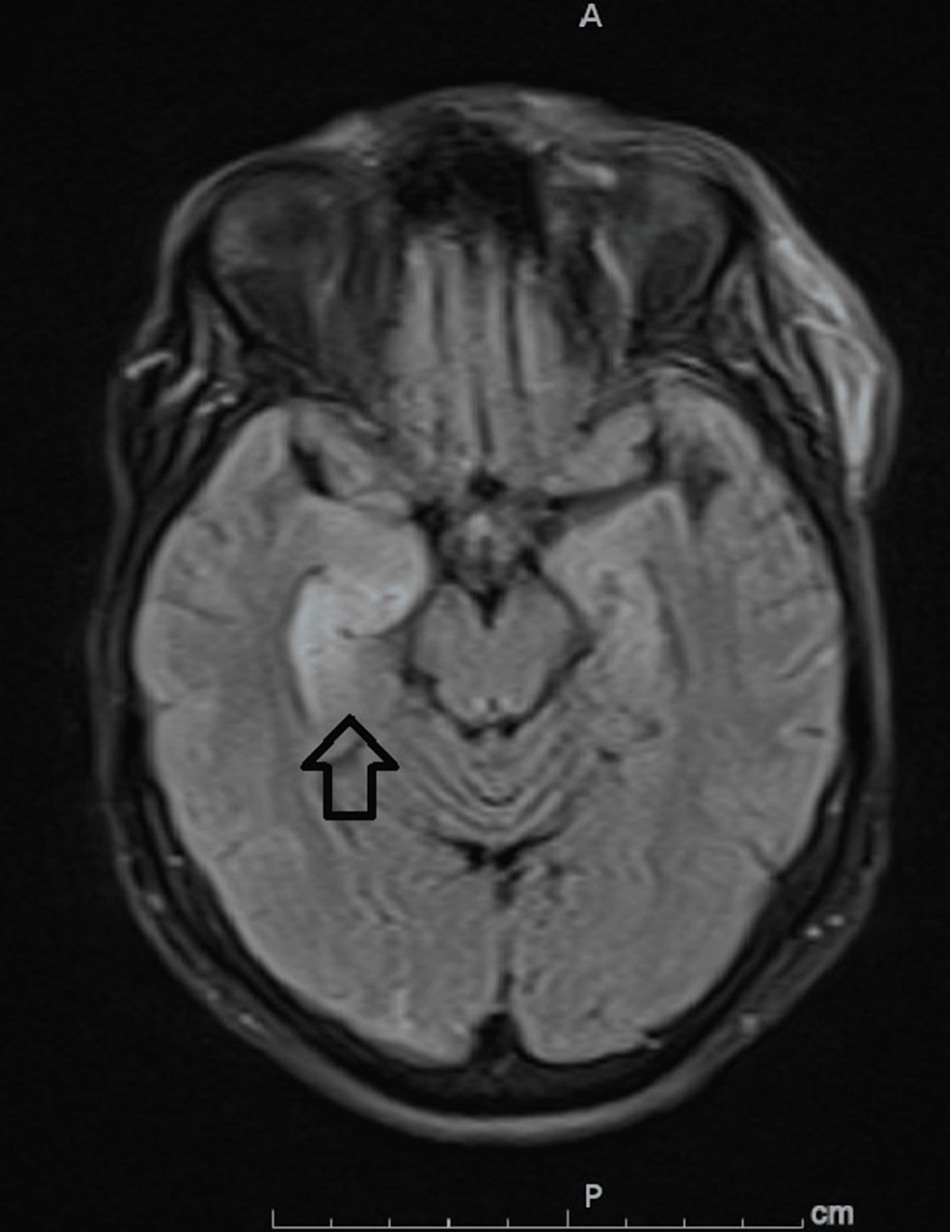
MRI Brain FLAIR MRI brain showing right-sided temporal lobe hyperintensity (arrow).

She presented one month later with persistent postural dizziness, progressively increasing frequency of abnormal left-sided facial and upper extremity movement, and recurrent falls. In ED, vital signs included a temperature of 37.1^o^C, heart rate of 92 beats per minute, respiratory rate of 16 cycles per minute, blood pressure of 160/80 mmHg, and SpO_2_ of 99% on room air. Physical exam was again limited by poor cooperation. However, it showed a limited orientation only to self, grossly normal muscle power, and deep tendon reflexes but with up-going plantar reflex on the left side. She limited examination of memory and concentration in cooperation. Otherwise, the exam was unremarkable. Basic lab work showed hyponatremia of 129 mmol/L. No evidence of infection was noted, and the trauma screen was remarkable, only for a small isodense subdural hematoma, subarachnoid hemorrhage, and left frontal cephalohematoma with no mass effect (Figure 2). Antiepileptic medication was started. Mild orthostatic hypotension was also noted and has failed to respond to fluid challenges and improved with midodrine. EEG was also unremarkable for epileptic activity and showed diffuse, generalized slowing with intermittent rhythmic delta slowing. A repeat MRI brain showed a new curvilinear FLAIR hyperintensity in the medial right temporal lobe with linear enhancement (Figure 3). Empiric treatment for herpes encephalitis was pursued.

However, lumbar puncture (LP) with cerebrospinal fluid (CSF) analysis showed clear fluid, elevated white blood cells (WBC) of 15 ( reference 0-5 cells/mcl), primarily lymphocytes, elevated protein of 97 mg/dl ( reference 12-60 mg/dl) and normal glucose level at 55 mg/dl. CSF culture was unyielding. Meningitis/encephalitis panel including Cryptococcus neoformans, cytomegalovirus, enterovirus, E.Coli K1, Haemophilus influenzae, Herpes simplex virus type 1,2 and 6, human parechovirus, Listeria, Neisseria, Streptococcus and Varicella zoster virus were all negative. However, oligoclonal bands and IgG index were not obtained. Despite escalating doses of antiepileptic medication, evidence of progressive frequent generalized subclinical electrographic seizure suggestive of multifocal cortical hyperexcitability was noted on subsequent EEG monitoring. She was started on a second antiepileptic medication. Focal slowing over the right temporal lobe was also seen on EEG. On telemetry monitoring, episodes of sinus bradycardia were noted. 

**Figure 4:**
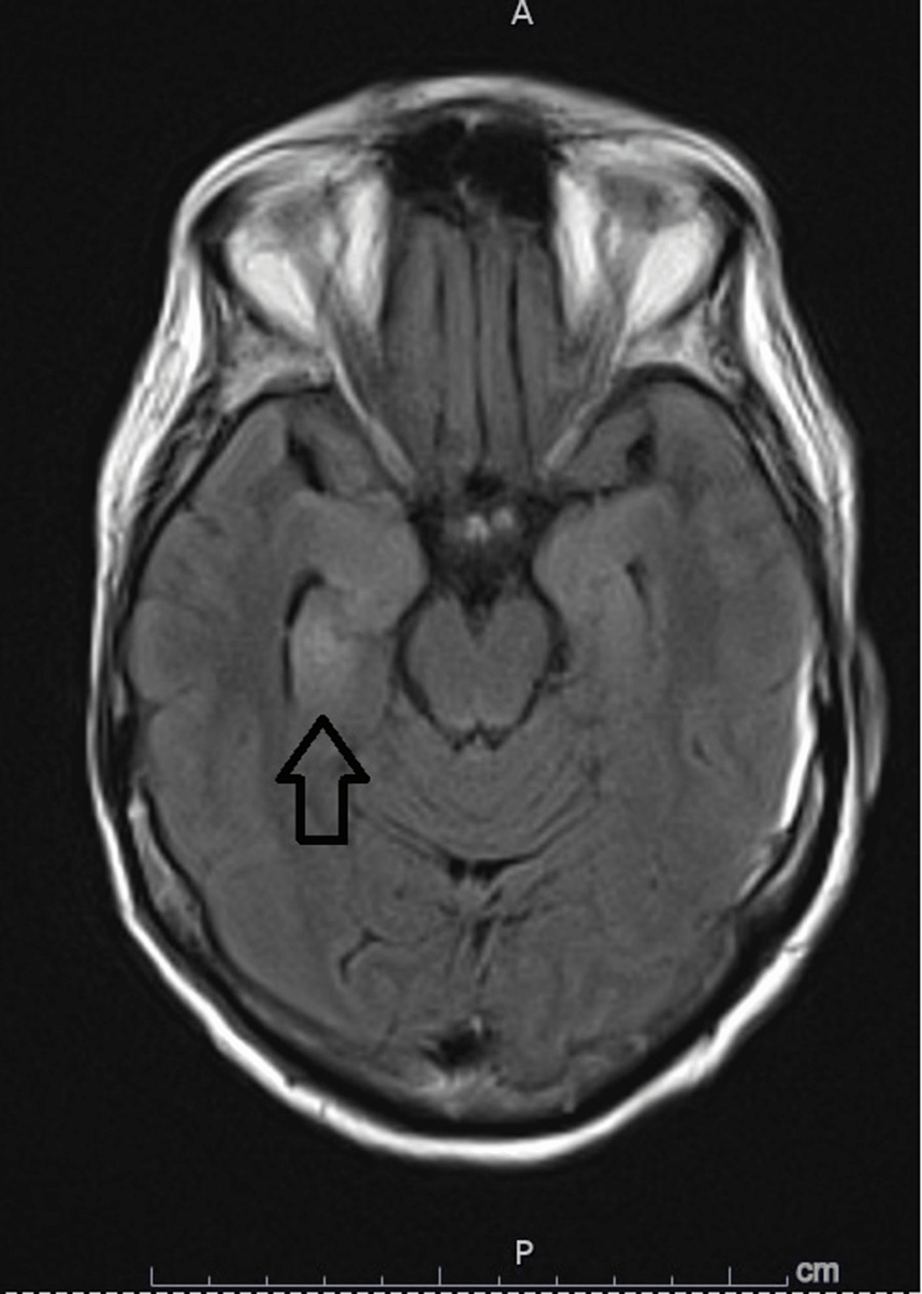
MRI Brain FLAIR MRI brain after IVIG and corticosteroid therapy showing evidence of improving right-sided temporal lobe hyperintensity (arrow).

Hyponatremia persisted despite fluid restriction and oral salt supplementation and ranged between low 120s to low 130s mmol/l. Autoimmune and paraneoplastic encephalitis were suspected, but cancer screening was unyielding. The paraneoplastic panel was negative for Anti-Yu, Anti-Ri, and Anti-Hu antibodies. Extensive autoimmune workup was also unyielding. The autoimmune encephalitis panel in the CSF was reactive to Anti-LGI1 IgG antibody at a titer of 1:70 ( negative if less than 1:10 ) but negative for Anti-NMDR, TPO, Anti-Ma2, Anti-GAD, Anti-NMO, Anti-MOG, Anti-DPPX, Anti-CASPR2 antibodies. Anti-LGI1 antibody was not detected in the serum. She was treated with intravenous immunoglobulin (IVIG) for 10 days and one gram daily of methylprednisolone for five days. The level of consciousness gradually improved, and a repeat MRI brain showed no new enhancement foci (Figure 4). 

Significant clinical improvement was evident at the end of one month of hospitalization. The seizure clinically improved with only a few faciobrachial dystonic seizures. She was discharged home, and a follow-up one month later showed more clinical improvement in memory, orientation, and concentration.

## Discussion

Leucine-rich glioma inactivated 1 (LGI1) antibody encephalitis is an autoimmune limbic encephalitis. Unlike other autoimmune encephalitides, Anti-LGI1 encephalitis is seldom a paraneoplastic process [5]. It accounts for 11.2% of all cases of autoimmune encephalitis [4]. VGKC encephalitis is a recently recognized autoimmune condition with antibodies against components of the VGKC protein complex. Voltage-gated potassium channels (VGKC) are important to regulate both central nervous system (CNS) and peripheral nervous system (PNS) excitability. VGKC exists in two types, LGI1 and CASPR2. LGI is a glycoprotein released from the presynaptic terminal to interact with presynaptic ADAM metalloproteinase domains 22 and 23, inhibiting signal transduction across synapses. Anti-LGI1 antibody inhibits this process and thus induces neuronal excitability [6]. 

The hallmark of this encephalitis is frequent multifocal seizure with different semiologies, which has poor prognostic significance [7]. It is estimated that 20-40% of those with Anti-LGI1 encephalitis have faciobrachial dystonic seizures (FBDS), which are short-lasting, usually for seconds, dystonic upper limb with ipsilateral facial contraction, which can also affect the lower limb [8]. The nature of FBDS is debated as dystonia or epileptic seizures [9]. FBDS usually precede cognitive decline and correlate with basal ganglia lesions [10]. Recognizing FBDS before other clinical manifestations with immunotherapy can help reduce long-term complications, including hippocampal atrophy and persistent memory impairment [11]. Cognitive decline is the acute or subacute onset and mainly affects memory, especially recently [10]. Mental symptoms are the main manifestation [5]. These can include agitation, anxiety, paranoia, personality, and behavioral changes, hallucinations, and impulsive behavior [11]. 

Brain MRI usually reveals bilateral T2 and FLAIR hyperintensities in the temporal lobes. The basal ganglia, insula, and hippocampus can also be affected [12]. MRI, as well as CSF analysis, can be normal. Anti-LGI1 antibody sensitivity in the blood is higher than CSF [5]. It is reported that the CSF antibody titer is only 1/10 of the serum antibody titer. Antibody titer does not always carry prognostic significance [13]. It should be noted that Anti-LGI1 antibodies can also be seen in cases of Creutzfeldt Jakob disease [14].

Although it was initially thought that the CSF titer of LGI1 antibody is only 1 out of 10 in the serum, a more recent large observational study has reported that the sensitivity of LGI1 antibody in the CSF is higher (100%) than in the serum (92%). Our patient's positive antibody findings in the CSF but not the serum align with this study's result. This study also found a poor correlation between the LGI1 titer and clinical outcome and prognosis. This study's median LGI1 antibody disappearance was two years [13]. 

Hyponatremia is seen in 60% of those with Anti-LGI1 antibody autoimmune encephalitis. It is believed that it is related to the syndrome of the inappropriate release of antidiuretic hormone (SIADH), which might be related to the simultaneous expression of LGI1 in both the kidneys and the hypothalamus [6]. 

FBDS is the most responsive feature of immunotherapy. Cognitive decline improves more slowly [10]. It is suggested that early combined immunotherapy, including glucocorticoids, IVIG, and plasma exchange, provides a better prognosis than glucocorticoids alone [4]. Cyclophosphamide and rituximab can also be used in refractory cases [15]. Around 70% have a good prognosis at two years follow up, with a recurrence rate of 30%, mostly in the first six months [10,13].

## Conclusions

Among patients with rapid progression cognitive decline, frequent refractory clinical/subclinical seizures, cardiac arrhythmias, autonomic dysfunction, and refractory hyponatremia should hint toward Anti-LGI1 encephalitis. This allows for early immunotherapy, improves long-term outcomes, and prevents structural brain damage. The positive LGI1 antibody in the CSF but not the serum supports the recent evidence of higher LGI1 antibody sensitivity in the CSF than in the serum.
